# Pattern and Outcome of Dengue Fever in a Pediatric Tertiary Hospital: A Retrospective Report

**DOI:** 10.7759/cureus.14164

**Published:** 2021-03-28

**Authors:** Wajid Hussain, Mehmood Shaikh, Muhammad Hanif, Muhammad Ashfaq, Habib Ahmed, Bader-u- Nisa

**Affiliations:** 1 Pediatrics and Child Health, Jinnah Sindh Medical University, Karachi, PAK; 2 Neonatal Intensive Care Unit, Jinnah Sindh Medical University, Karachi, PAK

**Keywords:** dengue fever/complications, dengue fever, children, dengue related hospitalization

## Abstract

Introduction

For Pakistan, dengue has been established as a public health problem. With superimposed factors such as poor socioeconomic conditions, limited public health awareness, poor hygiene, and sanitation conditions, the situation has become more severe and complications have become frequent. Almost 90% of all infections occur in children of age less than 18 years. This is a three-year retrospective report of dengue fever in Southern Pakistan.

Methods

In this retrospective analysis, all records of patients admitted to the National Institute of Child Health, Karachi, from May 1, 2016, till April 30, 2019, diagnosed with dengue fever were recruited. Their demographic, clinical, and biochemical records were assessed. The outcome was also recorded. Data were entered and analyzed using Statistical Package for Social Sciences (SPSS) for Windows version 20.0 (IBM Corp., Chicago).

Results

Among 93 cases of dengue fever, there were 71 (76.3%) male and 22 (23.7%) female children. Their mean age was 5.7 ± 3.07 years. The mean duration from onset of disease to hospitalization was 4.2 ± 2.1 days. The mean platelet count was 47391.30 ± 41370.61 x 10^9^/L. Fever (100%) and abdominal pain (35.5%) were common presentations. Bleeding episodes were seen in 31% of children, rash in 15%, disseminated intravascular coagulation in 3%, and 1% developed pleural effusion. There were no mortalities; 87 (93.5%) were discharged and six (6.5%) children left against medical advice.

Conclusion

Fever, abdominal pain, bleeding episodes, and rash were common presentations. Hematological, hepatological, neurological, and pleural complications were not uncommon. The outcome of the disease was adequate and there were no mortalities.

## Introduction

Dengue is an acute febrile arboviral illness. It is mainly endemic in tropical and subtropical areas as its transmitting mosquito - Aedes genus - is primarily seen in both these areas. The average incubation period is seven days (three to 14 days) [1]. The spectrum of dengue ranges from a non-severe febrile disease to a severe systemic life-threatening illness [2].

World Health Organization (WHO), reported approximately 400 million cases of dengue fever annually and the majority of cases of dengue fever are reported from Asian countries [3]. Dengue is now endemic in Pakistan and a public health threat especially for children [4]. In Pakistan, superimposed factors such as poor socioeconomic condition, limited public health awareness, poor hygiene, and sanitation conditions resulting in a more severe form of dengue fever [5].

Dengue affects all age groups; however, 90% of dengue infections occur in children of age less than 18 years [6]. Children have a 15-times higher risk of dying during a second episode compared to adults [7]. Fever remains the most common presentation of dengue in children followed by vomiting, and abdominal pain [8]. Complications include severe shock, disseminated intravascular coagulation (DIC), acute respiratory distress syndrome (ARDS), and hepatic and neurological involvement [9].

Older outbreaks, particularly from northern Pakistan have been reported in detail in the literature; however, there remains limited data depicting the situation in southern Pakistan [10-12].

## Materials and methods

For the retrospective analysis, all records of patients admitted at the National Institute of Child Health, Karachi, from May 1, 2016, till April 30, 2019, that were diagnosed with dengue fever were recruited. After taking permission from the hospital administration the records were recruited.

Data collection

Definition

Dengue fever: Dengue fever is an acute febrile illness defined by the presence of fever and two or more headache, myalgia and/or bone pain, arthralgia, retro-orbital or ocular pain rash, hemorrhagic manifestations (e.g., positive tourniquet test, petechiae, purpura/ecchymosis, epistaxis, gum bleeding, blood in emesis, urine, or stool, or vaginal bleeding), or leukopenia.

Dengue hemorrhagic fever: It is characterized by the evidence of plasma leakage due to increased vascular permeability, such as hemoconcentration, pleural effusion, or ascites in addition to the manifestation of dengue fever.

Dengue shock syndrome: It consists of dengue hemorrhagic fever with marked plasma leakage that leads to circulatory collapse (shock) as evidenced by narrowing pulse pressure or hypotension.

Admission Criteria

All those patients who visited the hospital, with a history of fever and or vomiting or rashes and according to hospital policies sick enough requiring IV fluids.

*Discharge Criteria* 

All dengue non-structural protein 1 (NS1) antigen or dengue serum immunoglobulin M (IgM) antibodies against dengue positive whose platelets were more than 50 thousand, defervescence of fever for the last 24 hour, and child can take per oral without vomiting were included in the discharge criteria.

Exclusion Criteria

Patients with positive blood culture for any bacterial pathogen: Patients concomitant having B thalassemia major, idiopathic thrombocytopenic purpura, acute leukemia, and aplastic anemia.

A semi-structured questionnaire was filled for data extraction. The variables were age and gender of the child, date of onset of symptoms, date of hospital admission, clinical signs and symptoms and duration of hospital stay, platelet count, dengue non-structural protein 1 (NS1), and dengue immunoglobulin M (dengue IgM). The outcome was classified as discharge, death, and left against medical advice (LAMA).

Data analysis

All data were entered and analyzed using Statistical Package for Social Sciences (SPSS) for Windows version 20.0 (IBM Corp., Chicago). Continuous variables, such as age, duration of onset of symptoms, duration of hospital stay, platelet count were presented as mean and standard deviation (SD) was calculated. Categorical variables, such as gender, clinical sign, and symptoms, dengue (IgM) positive, and outcome, were presented in frequency and percentages.

## Results

Among 93 cases of dengue fever-related hospitalizations, there were 71 (76.3%) male children. The mean age of the children was 5.7 ± 3.07 years (range: six months to 12 years). There were 15 (16.1%) hospitalizations in 2019, 31 (33.3%) in 2018, and 47 (50.5%) in 2017 making an estimated 31 dengue-related hospital admissions annually. Out of 93 patients, 64 (68.8%) were dengue fever, 26 (28%) were dengue hemorrhagic fever, and three (3.22%) dengue shock syndrome.

The mean duration from onset of disease to hospitalization was 4.2 ± 2.1 days (range: two to eight days). Demographic characteristics of enrolled patients are summarized in Table 1.

**Table 1:** Demographic characteristics of the patients (N=15)

Patient characteristics	Frequency (%)
Gender	
Male	71 (76.3%)
Female	22 (23.7%)
Age in years	
≤ 5	45 (48.4%)
6-10	40 (43.0%)
≥ 11 years	8 (8.6%)
Mean ± SD	5.7 ± 3.07
Mean duration from onset of symptoms to hospital admission in days	4.2 ± 2.1

Table 2 summarize the clinical and biochemical profile. All children had a fever and 29 (31.2%) had bleeding from the nose, mouth, ears, or petechiae. Four (4.3%) children had hematuria. The mean platelet count was 47,391.30 ± 41,370.61 x 10^9^/L (range: 2000-251,000 x 10^9^/L).

**Table 2:** Clinical characteristics of the patients (N=15) D IgM: dengue immunoglobulin M; DNS1: dengue non-structural protein 1

Patient characteristics	Frequency (%)
Signs and symptoms	
Fever	93(100%)
Generalized rash	14 (15.1%)
Abdominal pain	33(35.5%)
Joint pain	17 (18.3%)
Vomiting	11(11.8%)
Complications	
Jaundice	9(9.6%)
Bleeding	29 (31.2%)
Petechiae	12 (12.9%)
Hematuria	4 (4.3%)
Disseminated intravascular coagulation	3 (3.2%)
Generalized/pedal edema	3(3.2%)
Pleural effusion	1 (1.1%)
Fits	1 (1.1%)
Laboratory tests	
Mean platelet count x10^9^/L at admission	47,391.30 ± 41,370.61
Only D IgM positive	34 (36.5%)
Both DNS1 and D IgM positive	12 (12.9%)

Of all patients with dengue fever, 87 (93.5%) were discharged and six (6.5%) children left against medical advice. No death was reported.

## Discussion

In this analysis, it was found that the proportion of 50.5% hospitalizations due to dengue were high in 2017. Fever, abdominal pain, and joint pain were common presentations. Bleeding, petechiae, and jaundice were common complications. There was one case of pleural effusion and fits due to neurological involvement in each. There were no mortalities.

Although a lot of literary work is being done to document the burden of dengue in both endemic and non-endemic countries, the empirical data governing the pediatric population is still scarce. A large multi-center study from India reported that 60% of children age five to 10 years had been infected with dengue at least once (immunoglobulin G {IgG} seroprevalence positive). Upon multivariate analysis, the type of housing and household water storage system were significant associations with seropositivity [13]. Reports from Passive Dengue Surveillance System (PDSSS) in Puerto Rico reported more than 4000 cases of dengue in children of age <18 months. Of all laboratory positive cases, 58% required hospitalization and 33% of hospitalized children had severe dengue [14]. In a five-year-long study from India examining the blood samples of suspected dengue patients age 15 years or less, 25% were positive for dengue. They were more common in male children (58%) and children age 10-<15 years (46%) [15].

Despite the high prevalence and increasing severity with every outbreak, there still is lesser data explaining the characteristics and outcomes of hospitalized severe cases of dengue as compared to the epidemiological perspective of the illness. In a large cohort conducted across 10 Asian and Latin American countries, 10% of all febrile episodes were serologically proven to be dengue; of these 11-19% were severe enough to require hospitalization [16]. In almost all of these studies, fever, abdominal pain, vomiting, and skin rash were the common clinical presentation of dengue fever in children. Thrombocytopenia was also extremely common [7,8]. The bleeding presentation was commonly reported in the form of petechiae, nose bleeds, hematuria, hemoptysis, bleeding gums, hematemesis, melena, menorrhagia, and subconjunctival hemorrhages [6,9,17].

Among our children with dengue, only IgM was positive in 37%, and both IgM and DNS1 in 13%. Our results are on the lower end. Comparatively, in Afroze et al., 87% of children were DNS1 positive and 15% were IgM positive [7]. IgM was positive 41%, IgG in 24% while in 31 % of the children population both were found positive [18]. The mean duration from onset of symptoms to hospital admission in our children was four days which means that they were transitioning from acute to sub-acute and late phase of the disease. DNS1 alone has been seen to be sufficient in the acute phase of the disease. None of our children had DNS1 alone positive. A positive combination of DNS1 and IgM is highly sensitive in diagnosing both acute and late phases of the disease [19].

The outcome of children hospitalized with dengue was very promising in our study. Only a few had severe and life-threatening complications including DIC and pleural effusion and there were no mortalities. Hepatorenal and neurological complications, respiratory distress, DIC, and severe shock have been reported commonly in the literature and comprise of the common causes of death in these children [9,10,18]. In suspecting severe dengue and studying disease progression, it is essential to comprehend the warning signs. Hemodynamic instability is a crucial warning sign in these children. Lethargy presented 72 hours before the onset of shock and dyspnea however 48 hours before in children with severe disease. Lethargy (adjusted odds ratio {ORa}: 9.15), dyspnea (ORa: 8.24), and abdominal pain (ORa: 6.78) independently correlated with the severity of dengue in these children [20].

Limitations

We identify key study limitations and suggest directions for future research on this topic: (1) this is a retrospective study design and review of files. Prospective studies will be helpful for clinical spectrum; (2) seasonal variations have not been considered. A longer duration of study is required to interpret accurately for it; (3) a study comprised of a small number of patients larger sample may be a true representative.

## Conclusions

Ninety-three cases of dengue fever were admitted over the three years period. Fever, abdominal pain, rash, and bleeding episodes were common presentations. Hematological, hepatological, neurological, and pleural complications were not uncommon. The outcome of the disease was adequate and there were no mortalities.
